# Application of evolutionary algorithms to design economic flat slab buildings based on the intended function

**DOI:** 10.1038/s41598-024-58763-8

**Published:** 2024-04-17

**Authors:** Mohammed Rady

**Affiliations:** Construction and Building Engineering Department, College of Engineering and Technology, Arab Academy for Science, Technology and Maritime Transport (AASTMT), B 2401 Smart Village, Giza, 12577 Egypt

**Keywords:** Optimization, Reinforced concrete, RC, Excel solver, Genetic algorithm, Structural design, Civil engineering, Engineering

## Abstract

Numerous studies revealed optimization techniques' applicability in minimizing the costs of reinforced concrete buildings. However, the existing literature has narrowly focused on optimizing buildings with a single function, such as residential or office buildings, hindering the generalization of the results. This paper aims to bridge the gap between optimization and structural engineering by obtaining the minimum-cost design of flat slab buildings with different intended functions. In this context, the optimal designs of 120 alternatives were obtained, considering various spans (4–8 m), live loads (2–10 kPa), and concrete compressive strength (25–40 MPa). The optimization was executed using the evolutionary algorithm provided in Microsoft Excel’s Solver tool. The optimization model permits the utilization of drop panels to resist punching stresses developed from the slab-column interaction. The objective function is the cost of materials and labor involved in constructing floors and columns. The decision variables are the floor dimensions and column configurations in dimensions and reinforcement. The structural constraints were applied per the Egyptian design code (ECP203-2020). Eventually, guidelines were developed to help the designers choose the economic floor system and quantities of materials based on the building's intended function.

## Introduction

Reinforced concrete (RC) flat slab systems have been extensively used to construct residential and office buildings^1,2^. The absence of beams in flat slab systems enables flexibility in placing partition walls and pass services^3,4^. Moreover, eliminating sharp edges leads to better fire resistance and minimizes the risk of concrete spalling^5,6^.

Conventionally, the design of flat slabs involves assuming preliminary concrete dimensions of structural elements, estimating the required reinforcement, and checking that the ultimate limit state (ULS) and serviceability limit state (SLS) requirements provided by design codes are fulfilled^7,8^. This process is usually iterative, time-consuming, and non-economic as it is based on a trial-and-error approach^9,10^. For this reason, researchers, for decades, have been exploring different optimization techniques to minimize the overall cost of the structure^8,11–13^. The power of optimization stems from the ability to consider different combinations of decision variables and seek the most economical one in a reasonable time^14^.

Parametric studies were conducted in the literature to compare the design results of several floor systems. Rady and Mahfouz^15^ assessed the impact of the span and concrete strength on the optimal costs of residential buildings. The decision variables included the steel bars and concrete dimensions of columns and floors. For flat plates (FP) and flat slabs with drops (FD), they reported that low concrete strengths and spans of less than 5 m are sufficient to minimize the costs of such buildings. Whiteley et al.^16^ proposed a novel lightweight RC beam grillage floor system as an alternative to conventional flat slabs and optimized the overall structural volume for office buildings. They reported that the concrete and steel quantities for the proposed system were only 50% compared to regular flat slabs. Furthermore, they found that properly allocating the columns in multi-span cases could further reduce the material volume by over 50% compared to a standard layout.

Several studies have explored strategies to reduce the material usage and environmental impact of reinforced concrete floor systems in buildings. Trinh et al.^17^ focused on reducing the carbon emissions of FP office buildings with variable spans and heights using a Branch-and-Reduce technique. They recommended reducing the slab-to-column area ratio to achieve sustainable design results. The optimized buildings achieved up to 18% reduction in the overall embodied carbon compared to the conventional designs. Miller et al.^18^ analyzed the environmental impacts of flat slab office buildings with alternate slab configurations. They considered three slab systems: prestressed FP, RC FP, and RC FD. The quantities of construction materials were compared, given that the dimensions of columns and drop panels were fixed. Robati et al.^19^ computed the life-cycle costs of a benchmark Australian office building with two slab systems (FD and waffle slabs) and two concrete strength alternatives. They assumed that the spacing between columns was fixed to simplify the calculations. The material quantities, carbon emissions, and energy consumption were compared for different systems.

Jayasinghe et al.^20^ explored the impact of minimizing embodied carbon single-story flat slab office building by conducting a parametric analysis on the column spacing, concrete grade, column size, slab thickness, and reinforcement details. The results showed that the optimal designs coincided with minimal allowable slab thickness in most cases. Nevertheless, lower concrete grades could reduce carbon up to 12% even with thicker slabs. Ismail and Meller^21^ optimized the shape of ribbed slabs in multi-story residential buildings to minimize embodied energy from concrete and steel. A novel 3D shape parameterization and decoupled analysis approach was developed, resulting in slabs with 48–64% less embodied energy than flat slabs.

Other researchers evaluated different structural system configurations for multi-story concrete buildings. Elhegazy et al.^22^ conducted a parametric study on 72 building models with regular live loads, varying stories (5–50 floors), and grid spacing to determine the optimal gravity and lateral systems based on direct cost. For medium and high-rise with short spans, dual systems with solid slabs were most economical, while for low-rise with longer spans, shear walls with flat slabs were optimal. Jayasinghe et al.^23^ quantified the embodied carbon savings from various strategies like optimizing concrete grade, slab thickness, post-tensioning, using alternative slab types and novel thin-shell floors across different spans. Compared to conventional flat slabs, savings ranged from 12% (optimized design) to 65% (thin shells).

Computational design methods have also been explored for RC optimization. Eleftheriadis et al.^24^ presented a building information modeling (BIM)-enabled approach to automate steel reinforcement specification for flat slabs, enabling generation of efficient designs. Similarly, Huberman et al.^25^ developed an optimization framework combining structural and thermal analyses to compare life-cycle energy of residential flat slab roofs and vaulted forms with reduced material quantities, showing over 40% embodied energy savings and nearly 25% total life-cycle energy reduction potential.

While prior research has proven the applicability of optimization techniques for minimizing the construction costs and environmental impacts of flat slab building designs, these studies have narrowly focused on optimizing flat slab buildings for a single function, such as residential or office buildings. Consequently, the practical implications of the findings in the existing literature cannot be generalized to different types of buildings. To this end, this study addresses this gap by conducting a parametric investigation, considering different sets of spans, live loads, and concrete grades to cover the diverse building's intended functions like residential apartments, commercial offices, hospitals, and factories. Accordingly, the author developed a design optimization model using evolutionary algorithms (EA) to minimize the direct costs of labor and materials. The proposed model allows the utilization of drop panels to resist the excessive punching stresses developed from the slab-column connections. The optimal designs were recorded for two floor systems (FP and FD); each system was subjected to a set of live loads (2–10 kPa) and column spacings (4–8 m). Furthermore, the impact of enhancing the concrete compressive strength on the building cost was assessed by considering four concrete grades between 25 and 40 MPa. Eventually, guidelines were concluded to help the designer choose an adequate design alternative based on the intended function of the building. All the buildings were designed per the provisions of the Egyptian code for the design of concrete structures (ECP203-2020)^26^.

## Design methodology

The flat slab buildings considered in the current study were loaded per the Egyptian code for calculating loads (ECL)^27^. The dead load included the slab self-weight, the flooring load (1.5 kPa), and the wall load. The wall load was calculated by uniformly distributing a typical story's total weight of partition walls over the floor area. The live load was specified based on the building usage. Table 1 shows the recommended values of live loads by ECL based on the building type. Partial safety factors of 1.4 for dead loads and 1.6 for live loads were used in the load combination of gravity loads suggested by the ECL.

**Table 1:** Live loads according to the building types.

Building type	Live load (kPa)
Residential buildings	2–3
Offices	2.5–4
Office archives	5–10
Hospitals	2.5–4
Lecture rooms	4
Libraries	10
Laboratories	4
Hotels	2–4
Worship areas	4–5
Roofs	1–2
Garages	3–5

Flat slabs may be analyzed using any method that fulfills the ULS and SLS of the design code. Overall, the design assumptions and loading schemes provided by ECP203-2020 are close to other design specifications such as the American Code Institute (ACI) and British Standard Institute (BSI). However, some differences arise due to applied safety margins, quality control procedures for construction materials, and other code-specific provisions. Accordingly, structural concrete design codes define design limits for different RC members, such as concrete dimensions, reinforcement ratios, span-to-depth ratios, and deflection. The present study used the direct design method provided by ECP203-2020 in the analysis. This method offers an empirical procedure to obtain the design bending moments at the critical sections of the slab. While the direct design method may not accurately model variations in column and beam stiffness to the same degree as the equivalent frame analysis approach, it remains an acceptable methodology for structural design purposes. However, the following conditions outlined in ECP203-2020 should be satisfied to ensure that the bending moments at the critical sections are adequate^3,14^. First, the floor layout should consist of at least three continuous spans in each direction. Second, the rectangularity (i.e., ratio of the longer to the shorter span) should not exceed 1.3. Third, the maximum difference between successive span lengths in each direction should not exceed 10%. The complete details regarding the procedures and underlying assumptions have been comprehensively documented in a recent study^14^. All the subsequent equations are per ECP203-2020^26^.

First, the total moment ${{M}}_{{o}}$ on the slab is calculated as follows:

1 $${{M}}_{{o}} = \frac{{{w}}_{{s}}{{L}}}{8}{\left({L } - {2}{{b}}_{{c}}/{3}\right)}^{2}$$

where ${{w}}_{{s}}$ is the slab’s uniform load, *L* is the span, and ${{b}}_{{c}}$ is the column’s width.

**Figure 1:**
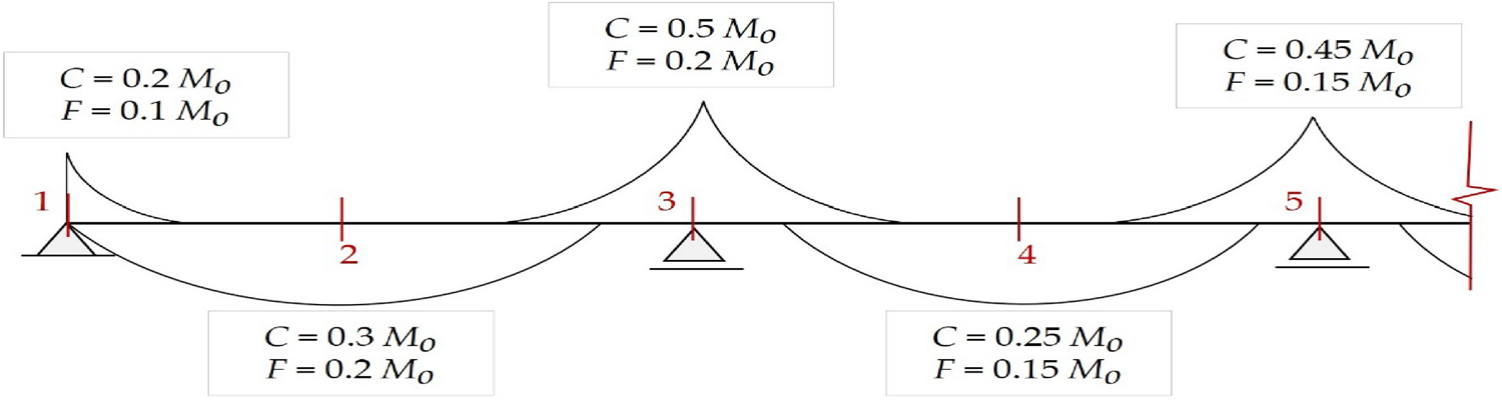
Percentages of total moment *M*_*o*_ distributed to critical sections of each strip. *C* is the column strip, and *F* is the field strip.

Second, *M*_*o*_ is distributed to the column and field strips in specific proportions recommended by ECP203-2020. The column strip is located at the column zone, whereas the field strip is located at the center, i.e., between the column strips. Figure 1 shows the empirical distribution of bending moments to the critical sections of each strip. The applied moment at any section shall not exceed the sectional flexural capacity. Accordingly, the required reinforcement per meter is calculated for each section based on the magnitude of the corresponding bending moment.

The chosen reinforcement area ${{A}}_{{s}}$ shall not be less than the minimum area of reinforcement ${{A}}_{{s,min}}$ provided by the ECP203-2020 in Eq. (2):

2 $${{A}}_{{s,min}} = {{max}}\left(\frac{0.6}{{{f}}_{{y}}}{b}_{{s}}{{{d}}}_{{s}}{, 0.0015}{b}_{{s}}{{{t}}}_{{s}}\right)$$

where ${{b}}_{{s}}$ is the width of the slab strip (1 m), ${{d}}_{{s}}$ is the effective depth of the slab, and ${{t}}_{{s}}$ is the slab thickness. The maximum spacing between the main reinforcement bars shall not exceed 200 mm. A top reinforcement mesh in both directions, not less than 20% of the main reinforcement, shall be provided to satisfy temperature and cracking requirements due to the large thickness of flat slabs.

ECP203-2020 accounts for both the cracked and uncracked stages when calculating deflections based on the effective moment of inertia method, which considers the transition from uncracked to fully cracked sections as the applied load. This approach is consistent with other widely recognized design guidelines, such as ACI and the Eurocode. The effect of creep was considered to account for the long-term deflection of the slab. Equation (3) gives the acceptable condition of the long-term deflection stated by ECP203-2020.

3 $${\Delta}_{{l}} = {\Delta}_{{dl}}\left({1+ \alpha}\right)+  {\Delta}_{{ll}} \le \frac{{L}}{{250}}$$

where ${\Delta}_{{l}}$ is the long-term deflection; ${\Delta}_{{dl}}$ and ${\Delta}_{{ll}}$ are the short-term deflections due to dead loads and live loads, respectively, *α* is a creep age factor.

Punching shear stresses developed from the slab-column interaction require special attention in flat slab buildings. The critical shear section is located at ${{d}}_{{s}}$/2 from the external column face. To resist the punching shear failure, the actual punching shear stress ${{q}}_{{up}}$ shall not exceed the concrete punching shear strength *q*_*cup*_ (Eq. (4)).

4 $${{q}}_{{cup}} = \min\left({0.8}\left(\frac{{\alpha}{{d}}_{{s}}}{{{b}}_{{o}}}{ + 0.2}\right)\sqrt{\frac{{{f}}_{{cu}}}{{\gamma}_{{c}}}}{, 0.316}\sqrt{\frac{{{f}}_{{cu}}}{{\gamma}_{{c}}}}{, 1.7 \; \text{MPa}}\right) \ge {{q}}_{{up}}$$

where ${{Q}}_{{up}}$ is the design shear load, ${{b}}_{{o}}$ is the critical shear perimeter, *α* is a coefficient based on the column location, and ${\gamma}_{{c}}$ is the safety reduction factor for concrete.

Providing drop panels above columns enhances the flat slabs in many ways. First, it increases the slab-column contact surface area allowing a better load distribution from slabs to columns. Second, it increases the slab’s negative flexural capacity as its effective depth increases. Third, it stiffens the flat slabs and reduces long-term deflection. The bottom reinforcement provided to drop panels is half that supplied to the column strip.

The columns were designed to sustain the factored axial load resulting from the slab and the column self-weight using the area method. The axial capacity of the columns was enhanced by adding vertical bars to the concrete cross-section. Stirrups were added to support the vertical bars and resist buckling. In addition, ECP203-2020 accounts for the bending moment transferred from slabs to the columns in the equivalent frame method by providing specific proportions of the bending moment at the column strip^14^.

**Figure 2:**
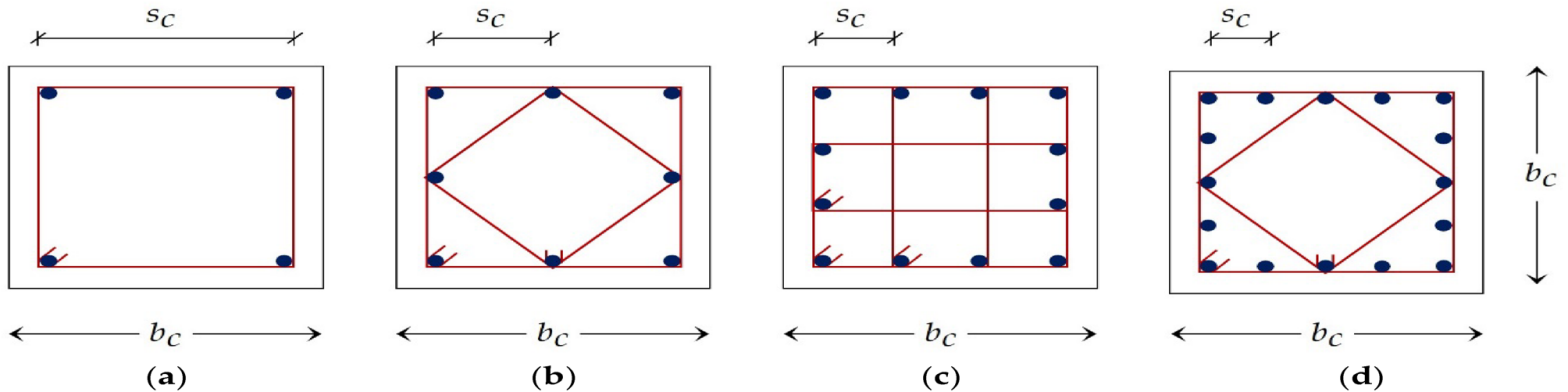
Column configurations: (**a**) alternative 1; (**b**) alternative 2; (**c**) alternative 3; (**d**) alternative 4.

Four possible configurations were suggested for the columns to guarantee that the spacing between two bars supported by ties ${{s}}_{{c}}$ does not exceed 0.25 m as imposed by ECP203-2020. Each configuration had a specific number of vertical bars and shape of stirrups based on the chosen column width ${{b}}_{{c}}$ (Fig. 2).

Equations (5), (6) give the allowable limits of the columns reinforcement as follows:

5 $${0.008}{{A}}_{{c}} \le { \mu } \le {\mu}_{{max}}$$

6 $${{V}}_{{s}} \, \ge \, {0.025}{{A}}_{{c}}$$

where *μ* is the ratio of the vertical reinforcement area to the concrete cross-sectional area, ${{V}}_{{s}}$ is the volume of lateral ties per meter, and ${{A}}_{{c}}$ is the cross-sectional area. The maximum reinforcement ratio ${\mu}_{{max}}$ equals 4, 5, and 6 for interior, edge, and corner columns, respectively.

**Figure 3:**
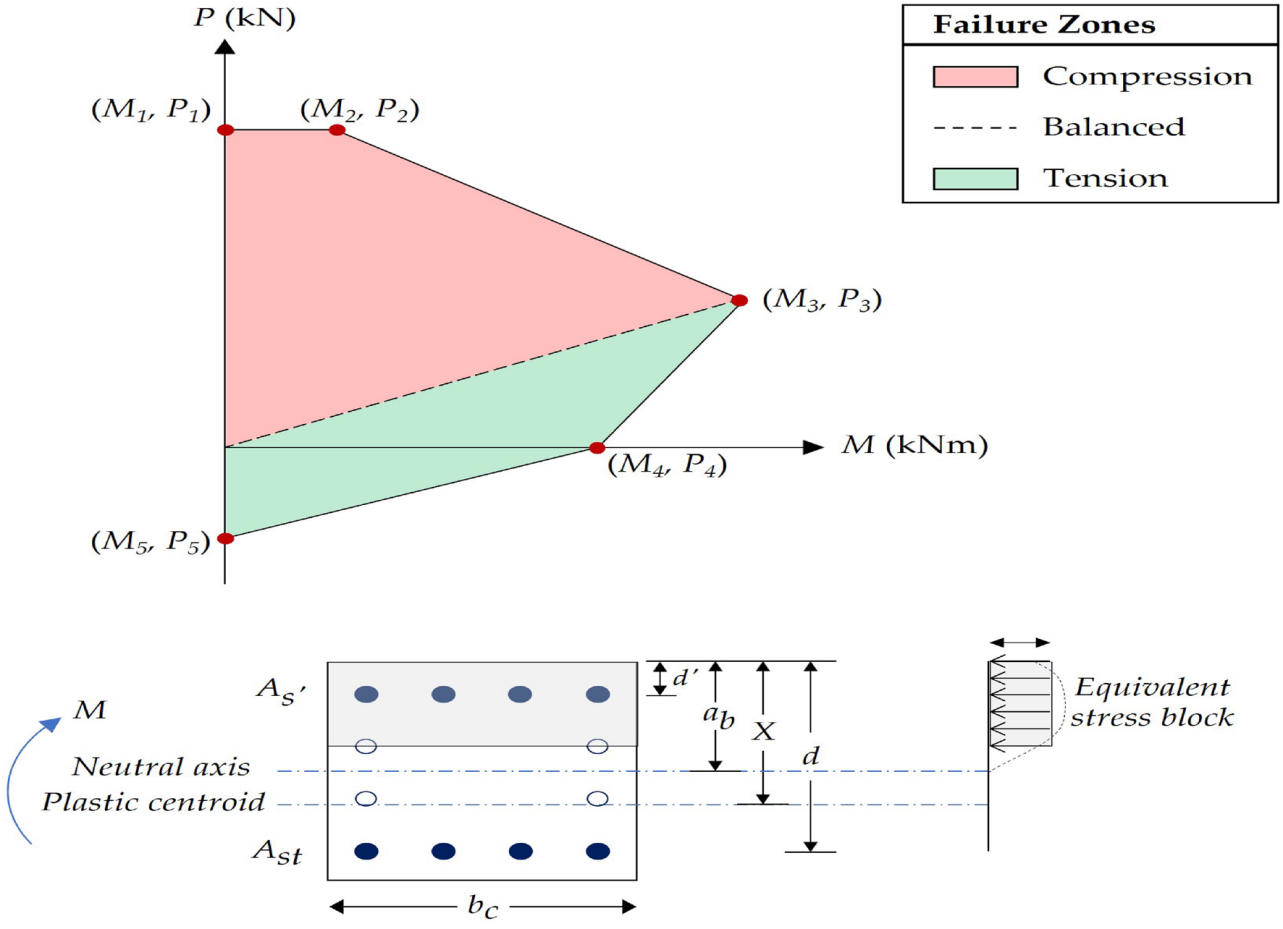
General interaction diagram and properties of column sections.

A simplified interaction diagram was constructed for each column to account for the unbalanced moment transferred from slabs to columns and consider the slenderness effect. The diagram was created by plotting five combinations of the section's nominal axial loads and bending moments. These combinations were calculated based on the various failure modes by changing the compression area of the column’s section. Subsequently, a point with a particular axial load and bending moment is plotted for each column. The column is considered safe if this point falls inside the interaction diagram boundaries. Figure 3 illustrates a typical interaction diagram the sectional properties for the columns. The equations required to plot the five combinations of loads and moments are given in Table 2.

**Table 2:** Interaction diagram equations.

Point	Description	Axial load		Bending moment	
1	Pure compression	${{P}}_{1} = {0.35}{{f}}_{{cu}}{(}{{A}}_{{c}}{ - }{{A}}_{{sc}}{) + }{0.67}{{f}}_{{y}}{{{A}}}_{{sc}}$	(7)	${{M}}_{1} = {0}$	(8)
2	Minimum eccentricity	${{P}}_{2}{ } = {{ P}}_{1}$	(9)	${{M}}_{2} = {{P}}_{2}\cdot {{e}}_{{min}}$	(10)
3	Balanced bending	${{P}}_{3} = {0.67}\frac{{{f}}_{{cu}}}{{\gamma}_{{c}}}{{a}}_{{b}}{b}_{{c}}{ } + \frac{{{f}}_{{y}}}{{\gamma}_{{s}}}{(}{{A}}_{{s^{\prime}}}{ - }{{A}}_{{st}}{)}$	(11)	${{M}}_{3}{ = 0.67}\frac{{{f}}_{{cu}}}{{\gamma}_{{c}}}{{a}}_{{b}}{{b}}\left({X - }\frac{{{a}}_{{b}}}{2}\right){ + }\frac{{{f}}_{{y}}}{{\gamma}_{{s}}}{{A}}_{{s^{\prime}}}\left({X - d^{\prime}}\right){ - }\frac{{{f}}_{{y}}}{{\gamma}_{{s}}}{{A}}_{{st}}\left({d - X}\right)$	(12)
4	Pure bending	${{P}}_{4} = {0}$	(13)	${{M}}_{4} = {{f}}_{{y}}{{{A}}}_{{st}}{(d - d^{\prime})}$	(14)
5	Pure tension	${{P}}_{5} = {{f}}_{{y}}{{{A}}}_{{sc}}$	(15)	${{M}}_{5} = {0}$	(16)

Descriptions of parameters: ${{A}}_{{sc}}$ is total reinforcement area; ${{f}}_{{y}}$ is the yield strength of the longitudinal reinforcement; ${{A}}_{{s^{\prime}}}$ is compressive bars area; ${{A}}_{{st}}$ is tensile bars area; ${{e}}_{{min}}$ is minimum allowable eccentricity; ${\gamma}_{{s}}$ is the safety reduction factor for steel reinforcement; ${{a}}_{{b}}$ is the distance of the equivalent rectangular stress block; *d* is the distance from the center of tensile bars to the extreme compression fibers of the section; ${d^{\prime}}$ is the distance from the center of compressive bars to the extreme compression fibers of the section; *X* is the distance from the plastic centroid to the extreme compression fibers of the section.

**Figure 4:**
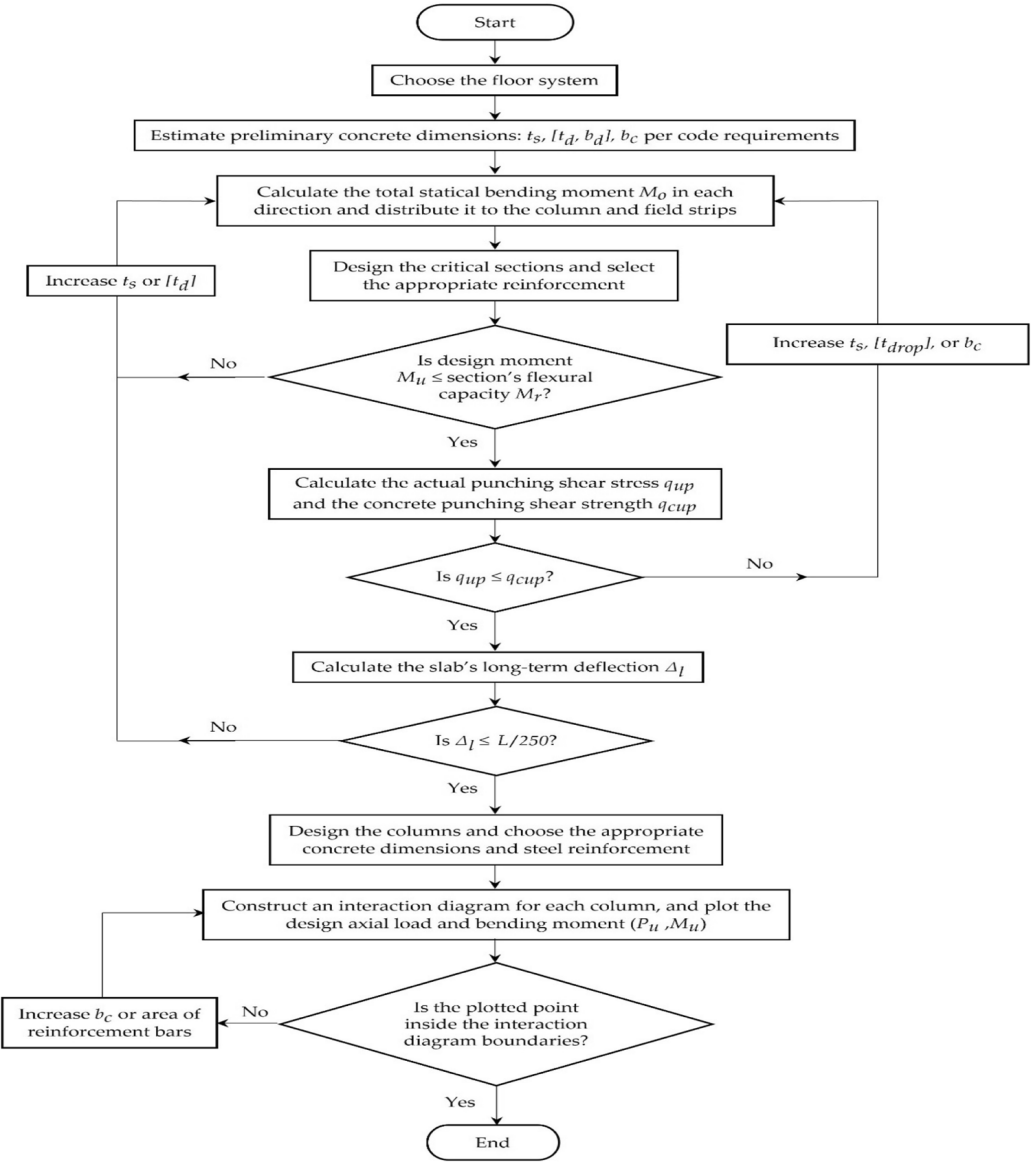
The flowchart of the typical design procedures of flat slabs and columns using the direct design method.

The flowchart in Fig. 4 summarizes the main steps of the conventional design of flat slabs and columns using the direct design method. The design process is iterative, and the number of possible feasible solutions is too large. Thus, a well-formulated optimization problem is necessary for a quick and economical design that fulfills the structural and practical requirements.

## Building description and assumptions

**Figure 5:**
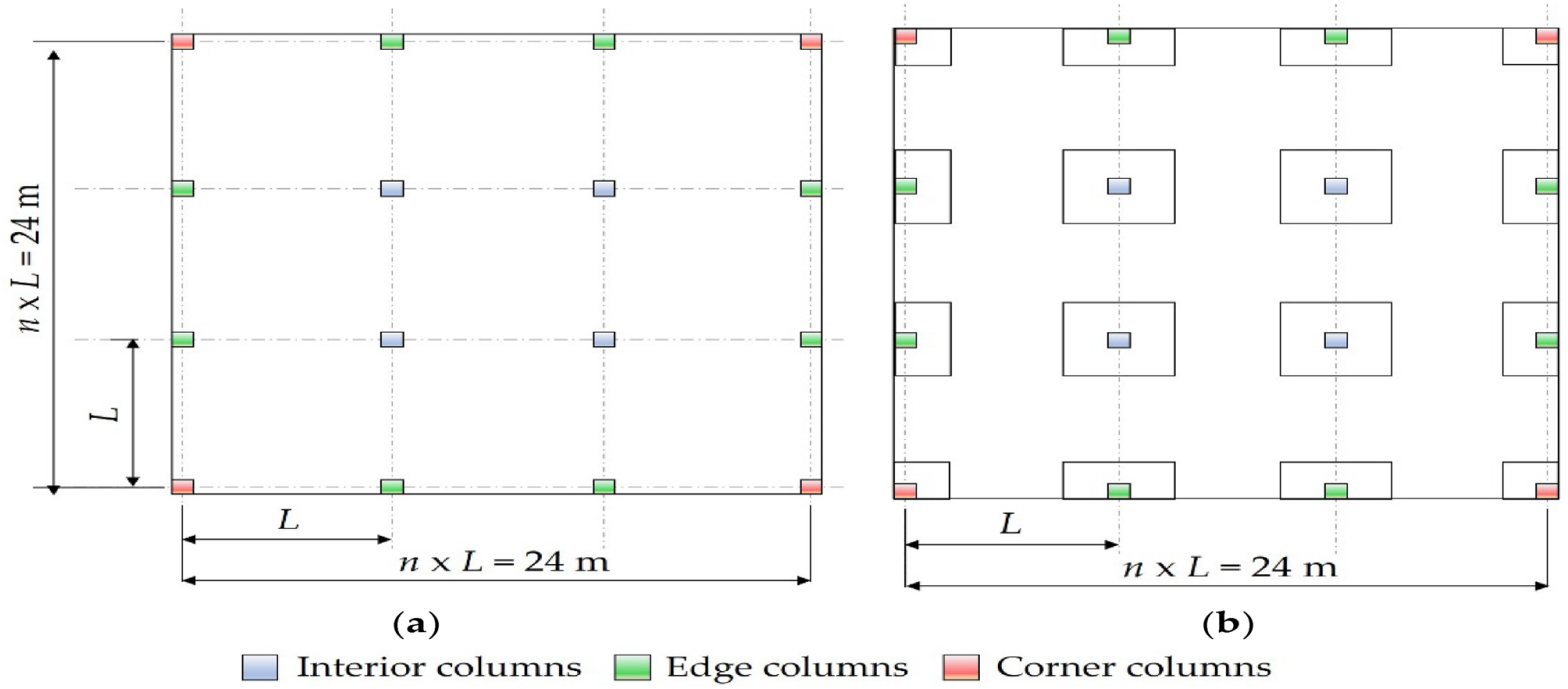
Identical floor layouts: (**a**) FP; (**b**) FD.

A four-story building with a square plan layout and a typical 3 m height was considered. The layout of the building was designed such that it can be divided into several span variants, having a side length of 24 m. Three typical cross-sections of square columns were designed based on their locations (i.e., intermediate, edge, and corner columns). Partition walls were distributed in both directions at the column grids. The optimization was performed for two floor systems: FP and FD. Figure 5 shows the proposed plan layout.

This study divides each floor into equal spans between 4 and 8 m to cover the typical span ranges in RC buildings. Moreover, live loads between 2 and 10 kPa were considered to account for the various functions of buildings. In total, fifteen variants of the flat slab building were made for each floor system. Each variant was independently optimized, and the optimal results were recorded.

## Problem formulation

### Objective function

In this study, the main goal of the optimization problem was to minimize the building’s total cost *T*. Therefore, the optimization problem can be generally stated as:

17 $${\text{Min}}{ T = f(x)}$$

subject to

18 $${{g}}_{{k}}\left({{x}}\right){ \le  1 \;\; k = 1, 2,..} {{n}}_{{k}}$$

19 $${{x}}_{{r}}^{{bl}} \le {{x}}_{{r}} \le {{x}}_{{r}}^{{bu}} \;\; { r = 1, 2,.. }{{n}}_{{r}}$$

where *x* is the decision variables set; *f(x)* is the cost function; ${{g}}_{{k}}\left({{x}}\right)$ is the inequality constraint function; ${{x}}_{{r}}^{{bl}}$ and ${{x}}_{{r}}^{{bu}}$ are the lower and upper bounds of the variable ${{x}}_{{r}}$, respectively; ${{n}}_{{k}}$ is the number of constraints; ${{n}}_{{r}}$ is the number of variables.

The objective function *T* is defined as follows:

20 $${T = }{{W}}_{{st}}{{{P}}}_{{st}}{ + }{{V}}_{{c}}{(}{{P}}_{{c}}+ { } {{P}}_{{f}}{)}$$

where ${{P}}_{{st}}$, ${{P}}_{{c}}$, and ${{P}}_{{f}}$ are the unit prices of steel, concrete, and formwork and labor, respectively; ${{W}}_{{st}}$ is the total weight of steel; ${{V}}_{{c}}$ is the total volume of concrete. These cost components were selected based on their predominant contribution to the overall cost of typical RC flat slab construction^3,10,14,15,28^. ${{P}}_{{st}}$ and ${{P}}_{{c}}$ were obtained from the Egyptian Ministry of Housing monthly bulletins reported on October 2022^29^. ${{P}}_{{f}}$ was considered based on the average prices obtained from multiple Egyptian construction sites. Table 3 shows the unit prices of the cost components utilized in this study.

**Table 3:** Unit prices of materials and labor.

Material	Strength (MPa)	Price (USD/unit)	Unit
Concrete	25	54.8	m^3^
	30	57.8	m^3^
	35	60.8	m^3^
	40	63.8	m^3^
Mild steel	240	920.0	ton
High tensile steel	350	920.0	ton
Formwork and labor	–	37.5	m^3^

### Decision variables

**Figure 6:**
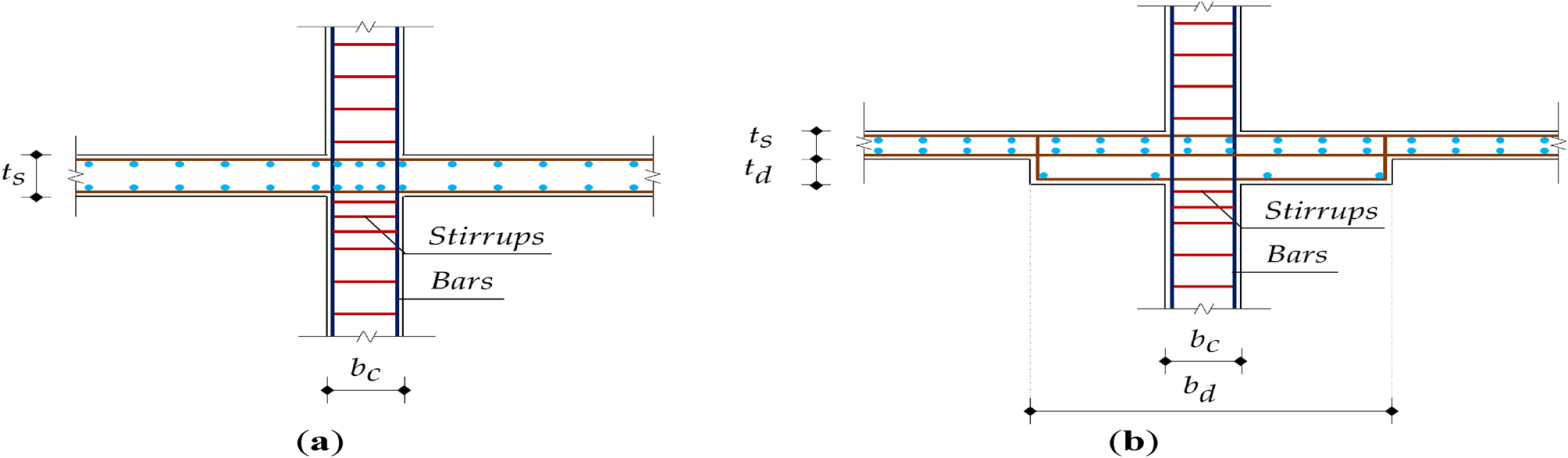
Decision variables: (**a**) flat plate (FP); (**b**) flat slab with drop (FD).

Figure 6 shows the decision variables of each floor system. Table 4 presents the lower and upper bounds for each variable. The upper bounds of the variables were reasonably selected to limit the search space of the optimization problem based on the design practice. The lower bounds of the variables were chosen to satisfy the minimum requirements imposed by ECP203-2020.

**Table 4:** Design variables of each floor system.

Floor system	Decision variable	Bounds	
FP	Slab thickness ${{t}}_{{s}}$	${\max(0.15 \; {\text{m}}, }{{L}}{/}{32}{) \le }{{t}}_{{s}}{ \le 0.34 \; {\text{m}}}$	(21)
	Column width ${{b}}_{{c}}$	${\max(0.3\; {\text{m}}, }{{L}}{/20) \le }{{b}}_{{c}}{ \le 1.2 \; {\text{m}}}$	(22)
	Column bar diameter ${\phi}_{{c}}$	${12 \; {\text{mm}} \le }{\phi}_{{c}}{ \le 25 \; {\text{mm}}}$	(23)
	Number of column stirrups ${{n}}_{{s}}$	${\max(5, }{{1000/(15}{\phi}}_{{c}}{)) \le }{{n}}_{{s}}{ \le 12}$	(24)
FD	Slab thickness ${{t}}_{{s}}$	${\max(0.15 \; {\text{m}}, }{{L}}{/}{36}{) \le }{{t}}_{{s}}{ \le 0.34 \; {\text{m}}}$	(25)
	Drop panel thickness ${{t}}_{{d}}$	${{t}}_{{s}}{/4 \le }{{t}}_{{d}}{ \le }{{t}}_{{s}}{/2}$	(26)
	Drop panel width ${{b}}_{{d}}$	${{L}}{/3 \le }{{b}}_{{d}}{ \le }{{L}}{/2}$	(27)
	Column width ${{b}}_{{c}}$	${\max(0.3 \; {\text{m}}, }{{L}}{/20) \le }{{b}}_{{c}}{ \le 1.2 \; {\text{m}}}$	(28)
	Column bar diameter ${\phi}_{{c}}$	${12 \; {\text{mm}} \le }{\phi}_{{c}}{ \le 32 \; {\text{mm}}}$	(29)
	Number of column stirrups ${{n}}_{{s}}$	${\max(5, }{{1000/(15}{\phi}}_{{c}}{)) \le }{{n}}_{{s}}{ \le 12}$	(30)

The variables were restricted to firm values to fulfill the construction requirements. The practical increments for concrete dimensions were: (i) 20 mm for the slab thickness ${{t}}_{{s}}$, (ii) 20 mm for the drop panel thickness ${{t}}_{{d}}$, (iii) 50 mm for the drop panel width ${{b}}_{{d}}$, and (iv) 25 mm for column width ${{b}}_{{c}}$. The bar sizes were restricted to those available on the market: 8, 10, 12, 16, 18, 22, and 25 mm. These increments can be adjusted based on the formwork dimensions and the commercial bar diameters. The minimum longitudinal bar diameters allowed for slabs and columns were 10 mm and 12 mm, respectively. The bar diameter used for the column stirrups was 8 mm. High tensile steel was used for the longitudinal reinforcement of slabs and columns with a yield strength of 350 MPa, and mild steel was used for the stirrups of columns with a yield strength of 240 MPa. These grades were selected because they are the most common steel grades utilized in the Egyptian building sector^14^. It is worth mentioning that decision variables such as the concrete dimensions and reinforcement bar sizes were defined as continuous variables. Nevertheless, these variables were rounded, and the rounded values were incorporated in the objective function. This approach was recommended by several studies to fulfill the practical considerations^3,10,14,15^.

### Constraints

Constraints were established to satisfy the practical considerations and the structural requirements imposed by the ECP203-2020. Design constraints related to the slabs include the spacing limits of reinforcement, the maximum allowable deflection, the spacing between bars, and the allowable punching shear stresses developed from the column-slab connection. Design constraints related to columns include the axial resistance, the bending moment resistance, the minimum and maximum reinforcement ratio, and the minimum stirrups volume. These constraints were discussed thoroughly in “Design methodology” section.

## Optimization algorithm

**Figure 7:**
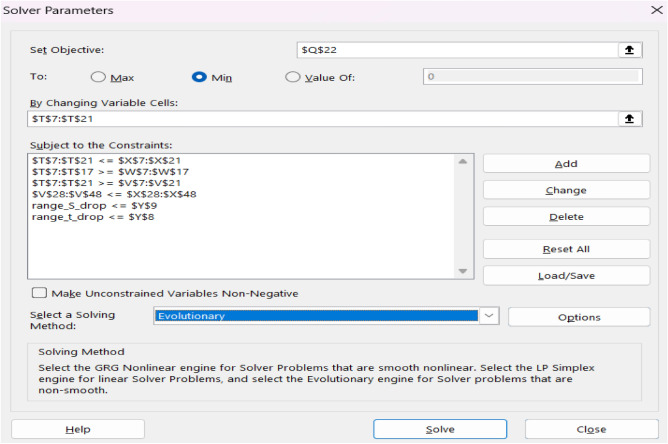
Solver parameters.

A mathematical model was constructed via Microsoft Excel 365 spreadsheets. The spreadsheets included the input data and the calculations regarding analysis, design, quantity surveying, and cost estimating of structural elements. The cost optimization was performed using the Excel Solver tool for its user-friendly interface and accessibility. Accordingly, problem formulation could be employed without the necessity of extensive programming knowledge. The Solver add-in allows the user to select the cells that include the objective function, adjustable values (i.e., decision variables), and constraints. Lower and upper bounds should be assigned to all decision variables to control the search space. When assigning the constraints, a user can choose different types (i.e., equality, inequality, integer, or binary) based on the mathematical problem. Figure 7 shows the Solver dialog box including the key parameters required for the problem formulation.

The Solver add-in provides three optimization methods: generalized reduced gradient (GRG), evolutionary algorithms (EA), and simplex. The simplex method can deal with linear problems only while the GRG and EA methods can deal with non-linear problems. The GRG method depends on calculating gradient or derivative information to iteratively converge on optimal feasible solutions. However, for problems involving objectives or constraints with discontinuities or non-smooth regions, the gradients cannot be numerically estimated correctly. This prevents GRG from yielding optimal solutions since it cannot properly account for the non-smooth areas where curvature information is undefined. On the other hand, EA relies on random sampling with localized deterministic searching to effectively explore wide search space^14,30^. Moreover, EA can accommodate discontinuous and non-smooth objective functions, unlike gradient-based techniques which require continuous differentiable formulas^31^. In the current investigation, the GRG method failed to converge to a reasonable global optimal solution because the model contains extensive non-smooth and discontinuous functions in the constraints and objective function. Accordingly, EA was employed to robustly deal with the complexity of the optimization model based on recommendations of previous studies^10,14,15,32^.

Given finite computational resources and time, it's practically impossible to exhaustively explore the entire solution space to guarantee finding every feasible solution. Instead, evolutionary algorithms aim to efficiently explore the solution space to find “good” solutions within a reasonable amount of time. The EA parameters to be adjusted include convergence value, mutation rate, population size, random seed, and maximum time without improvement^15,24,25^. Table 5 tabulates the Solver parameter values used in the present analysis. These values were chosen based on recommendations from previous studies^14,15,32^. According to the parameter tuning conducted by Rady and Mahfouz^15^, these values yield the best performance by exploring more possibilities, increasing the diversity of the population, while reducing the computational time.

**Table 5:** Parameters used in the solver tool for optimization.

Parameter	Value
Convergence	0.0001
Constraint precision	0.000001
Random seed	0
Population size	100
Mutation rate	0.075
Maximum time without improvement	60 s

**Figure 8:**
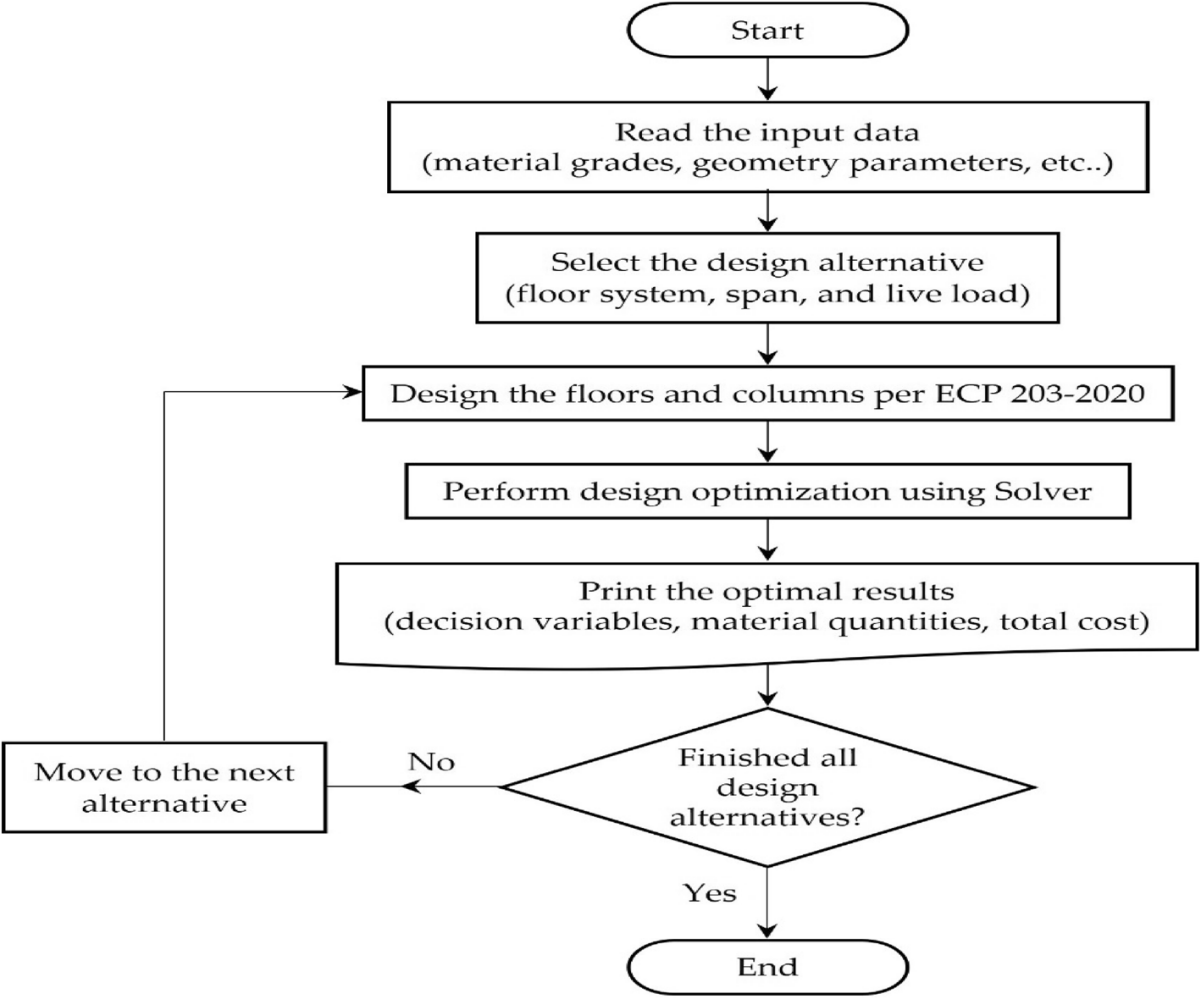
Computational flowchart of the developed optimization model.

Visual Basic for Applications (VBA) was used to automate the model. Repetitive tasks such as running the optimization tool, changing the spans and live loads, and constructing tables were programmed using VBA. This combination of spreadsheets, Solver, and VBA strongly enhanced the process, creating a robust environment for the optimization process. The computational flowchart in Fig. 8 summarizes the steps of the optimization model.

## Results and discussion

The optimal results from flat slab building design alternatives with ${f}_{cu}$ of 25 MPa are summarized in Table 6. These results include information about the concrete dimensions, reinforcement, quantities of construction materials, and total costs. The following subsections discuss the impact of different parameters on each floor system.

**Table 6:** Summary of optimal results for different span and live load variants.

Floor system	*L* (m)	*LL* (kPa)	Floor			Interior columns				Edge columns				Corner columns				Concrete volume (m^3^)		Reinforcement weight (ton)		Total cost (USD/m^2^)
			${{t}}_{{s}}$ (m)	${{t}}_{{d}}$ (m)	${{b}}_{{d}}$ (m)	${{b}}_{{c}}$ (m)	Bars (mm)	Stirrups (mm/m)	*μ* (%)	${{b}}_{{c}}$ (m)	Bars (mm)	Stirrups (mm/m)	*μ* (%)	${{b}}_{{c}}$ (m)	Bars (mm)	Stirrups (mm/m)	*μ* (%)	Floor	Columns	Floor	Columns	
FP	4	2	0.16	–	–	0.35	8T16	5Y8	1.31	0.30	4T16	5Y8	0.89	0.30	4T16	5Y8	0.89	92.16	14.83	7.83	2.24	33.22
		4	0.16	–	–	0.40	8T16	5Y8	1.01	0.35	8T16	5Y8	1.31	0.30	4T16	5Y8	0.89	92.16	19.34	8.34	2.82	35.68
		6	0.16	–	–	0.45	8T18	6Y8	1.01	0.35	8T16	5Y8	1.31	0.30	4T16	5Y8	0.89	92.16	22.36	8.90	3.29	37.82
		8	0.18	–	–	0.45	8T18	6Y8	1.01	0.35	8T16	5Y8	1.31	0.30	4T16	5Y8	0.89	103.68	22.20	8.95	3.29	39.72
		10	0.18	–	–	0.50	8T18	6Y8	0.81	0.40	8T16	5Y8	1.01	0.35	8T16	5Y8	1.31	103.68	28.03	9.38	3.47	41.61
	6	2	0.20	–	–	0.55	8T22	7Y8	1.01	0.45	8T18	6Y8	1.01	0.40	8T16	5Y8	1.01	115.20	16.22	10.45	2.30	41.42
		4	0.22	–	–	0.60	12T18	7Y8	0.85	0.50	8T18	6Y8	0.81	0.45	8T18	6Y8	1.01	126.72	19.60	11.20	2.38	45.12
		6	0.24	–	–	0.65	12T22	7Y8	1.08	0.50	8T18	6Y8	0.81	0.45	8T18	6Y8	1.01	138.24	21.01	11.82	2.91	49.03
		8	0.24	–	–	0.75	12T22	8Y8	0.81	0.60	12T18	7Y8	0.85	0.55	8T22	7Y8	1.01	138.24	29.24	13.04	3.65	53.48
		10	0.28	–	–	0.75	12T22	8Y8	0.81	0.55	8T22	7Y8	1.01	0.50	8T18	6Y8	0.81	161.28	26.36	13.25	3.50	56.80
	8	2	0.28	–	–	0.75	12T22	8Y8	0.81	0.60	12T18	7Y8	0.85	0.60	12T18	7Y8	0.85	161.28	17.87	13.73	2.21	54.16
		4	0.32	–	–	0.80	12T25	9Y8	0.92	0.60	12T22	7Y8	1.27	0.60	12T18	7Y8	0.85	184.32	18.44	15.23	2.91	61.44
		6	0.34	–	–	0.90	16T25	10Y8	0.97	0.70	12T22	8Y8	0.93	0.60	12T22	7Y8	1.27	195.84	22.88	15.69	3.51	65.69
		8	0.34	–	–	1.00	16T28	11Y8	0.99	0.75	12T22	8Y8	0.81	0.70	12T22	8Y8	0.93	195.84	27.82	17.29	3.94	69.72
		10	0.34	–	–	1.10	20T25	12Y8	0.81	0.85	16T22	10Y8	0.84	0.75	12T22	8Y8	0.81	195.84	34.23	18.81	4.44	73.99
FD	4	2	0.16	0.04	2.00	0.35	8T16	5Y8	1.31	0.30	4T18	5Y8	1.13	0.30	4T16	5Y8	0.89	97.92	14.83	8.15	2.39	34.90
		4	0.16	0.04	2.00	0.40	8T16	5Y8	1.01	0.35	8T16	5Y8	1.31	0.30	4T16	5Y8	0.89	97.92	19.34	8.69	2.82	37.17
		6	0.16	0.04	2.00	0.40	8T18	5Y8	1.27	0.35	8T16	5Y8	1.31	0.30	4T16	5Y8	0.89	97.92	19.34	9.61	3.20	39.24
		8	0.16	0.04	2.00	0.45	8T18	6Y8	1.01	0.35	8T16	5Y8	1.31	0.30	4T16	5Y8	0.89	97.92	22.36	10.19	3.29	40.80
		10	0.16	0.04	2.00	0.45	8T18	6Y8	1.01	0.40	8T16	5Y8	1.01	0.30	4T16	5Y8	0.89	97.92	24.49	10.92	3.32	42.34
	6	2	0.20	0.06	2.40	0.50	8T18	6Y8	0.81	0.45	8T18	6Y8	1.01	0.40	8T16	5Y8	1.01	120.73	14.90	11.40	1.92	42.99
		4	0.20	0.10	2.00	0.60	12T18	7Y8	0.85	0.45	8T18	6Y8	1.01	0.40	8T18	6Y8	1.27	121.60	17.67	12.28	2.35	45.67
		6	0.20	0.10	2.00	0.60	12T18	7Y8	0.85	0.50	8T18	6Y8	0.81	0.40	8T18	5Y8	1.27	121.60	19.26	13.82	2.37	48.41
		8	0.20	0.10	2.15	0.65	12T22	7Y8	1.08	0.50	8T18	6Y8	0.81	0.40	8T18	5Y8	1.27	122.60	20.84	15.61	2.90	52.53
		10	0.22	0.12	2.20	0.65	12T22	7Y8	1.08	0.50	8T22	7Y8	1.22	0.40	8T22	5Y8	1.90	136.01	20.69	15.70	3.52	55.79
	8	2	0.24	0.12	2.80	0.65	12T22	7Y8	1.08	0.60	12T18	7Y8	0.85	0.50	8T18	6Y8	0.81	146.71	15.37	15.47	2.02	53.89
		4	0.24	0.12	2.75	0.75	12T22	8Y8	0.81	0.60	12T18	7Y8	0.85	0.50	8T18	6Y8	0.81	146.41	16.92	17.65	2.05	57.62
		6	0.26	0.12	2.70	0.80	12T25	9Y8	0.92	0.65	12T22	7Y8	1.08	0.55	8T22	7Y8	1.01	157.63	19.59	19.26	2.93	63.82
		8	0.26	0.12	2.75	0.85	16T22	10Y8	0.84	0.65	12T22	7Y8	1.08	0.55	8T22	7Y8	1.01	157.93	20.50	21.71	2.94	67.95
		10	0.28	0.12	2.75	0.90	16T25	10Y8	0.97	0.70	12T22	8Y8	0.93	0.65	12T22	7Y8	1.08	169.45	24.07	23.07	3.51	73.45

### Ratios of floors and columns

Generally, the floors constituted the central portion of the concrete volume and reinforcement weight. In the case of FP alternatives, the floor’s concrete volume ranged between 79 and 90% of the total volume, and the floor’s reinforcement ranged between 73 and 86% of the total reinforcement. Similarly, in the case of FD alternatives, the floor’s concrete volume ranged between 80 and 91% of the total volume, and the floor’s reinforcement ranged between 75 and 90% of the total reinforcement^6,14,33^. These results are in line with previous studies, where they reported that floors are responsible for the highest construction costs. Hence, more effort should be directed to optimizing floors rather than columns.

Recent studies reported that steel constitutes a significant construction cost due to its high unit price^14,15^. Here, the reinforcement ratios for all columns ranged between 0.81 and 1.31%. Hence, the optimizer tended to reduce the reinforcement ratio to reduce the overall cost.

#### Effect of live load

**Figure 9:**
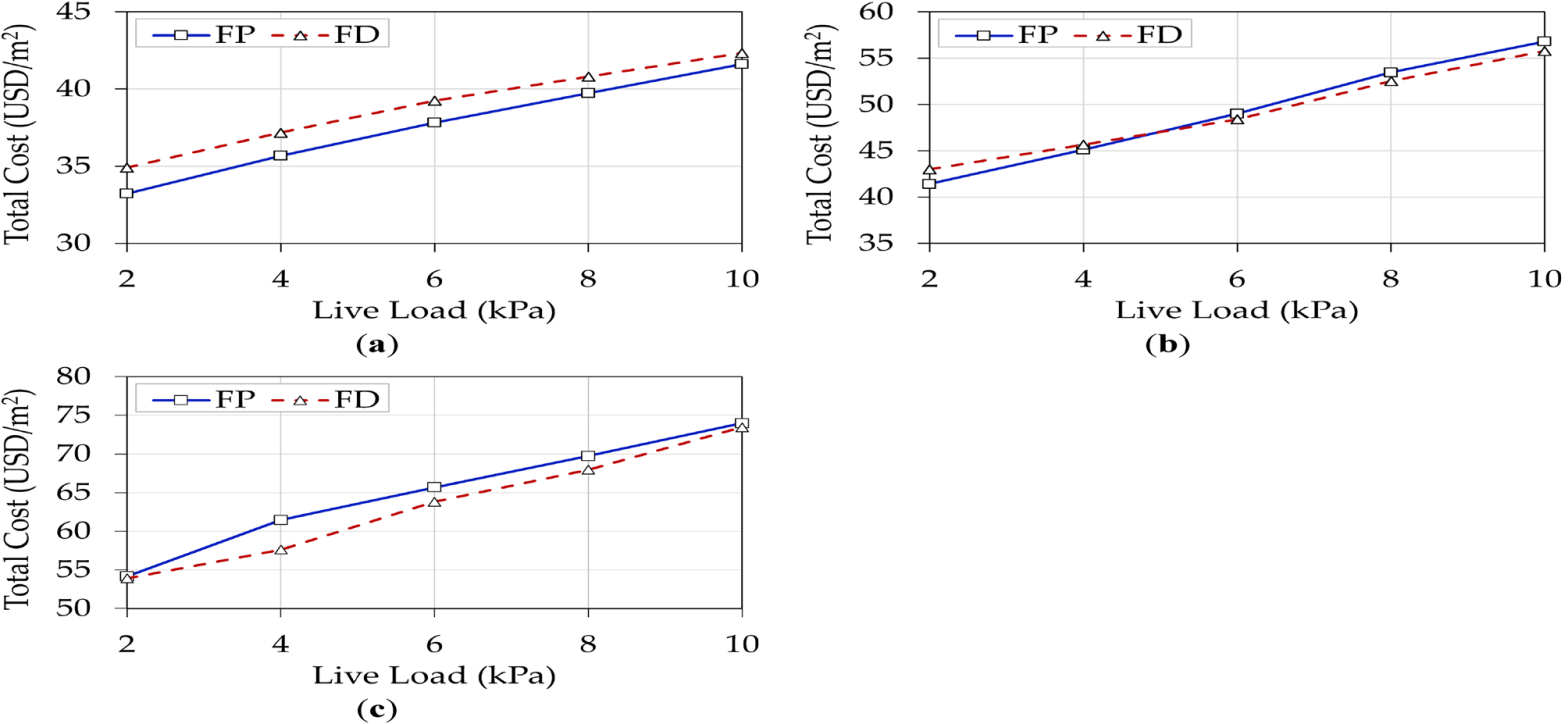
Effects of live loads on the total optimal cost for each floor system: (**a**) *L* = 4 m; (**b**) *L* = 6 m; (**c**) *L* = 8 m.

Figure 9 illustrates the impact of five live loads on the total optimal building costs with ${f}_{cu}$ of 25 MPa. The optimal results were generated for each floor system, considering three common column spacings (4, 6, and 8 m). The results show that increasing the live load for both systems increased the building’s total cost significantly.

A recent study reported that FP is cheaper than FD for spans up to 7.5 m^15^. However, the study was limited to residential buildings only. In the current study, this observation was confirmed for residential buildings up to 6 m only. As the span increased to 8 m, the optimal designs of both systems became almost equal. FP designs were cheaper than those of FD for buildings with a typical column spacing of 4 m, regardless of the building’s intended function. Table 6 shows that the value of ${{t}}_{{s}}$ was 0.16 m for all design alternatives with *L* = 4 m. This implies that drop panels are ineffective for low spans because the absolute minimum slab thickness was sufficient to resist the straining actions (flexure, punching shear, etc.) and deflection. As the live load increases, the costs of both systems become closer.

For buildings with a 6 m span subjected to low live loads, FP designs were cheaper than those of FD. As the live load exceeded 4 kPa, FD became cheaper. The maximum cost difference between the systems was 1.81% at 10 kPa. As the column spacing reached 8 m, FD was cost-effective under all considered live loads. This implies that the drop panels efficiently resisted the high punching stresses at the slab-column interactions. On the other hand, in the case of FP, ${{t}}_{{s}}$ significantly increased to resist the punching stresses, resulting in higher concrete volumes and amount of reinforcement. A similar conclusion was reached by Rady and Mahfouz^15^.

#### Effect of concrete grade

**Figure 10:**
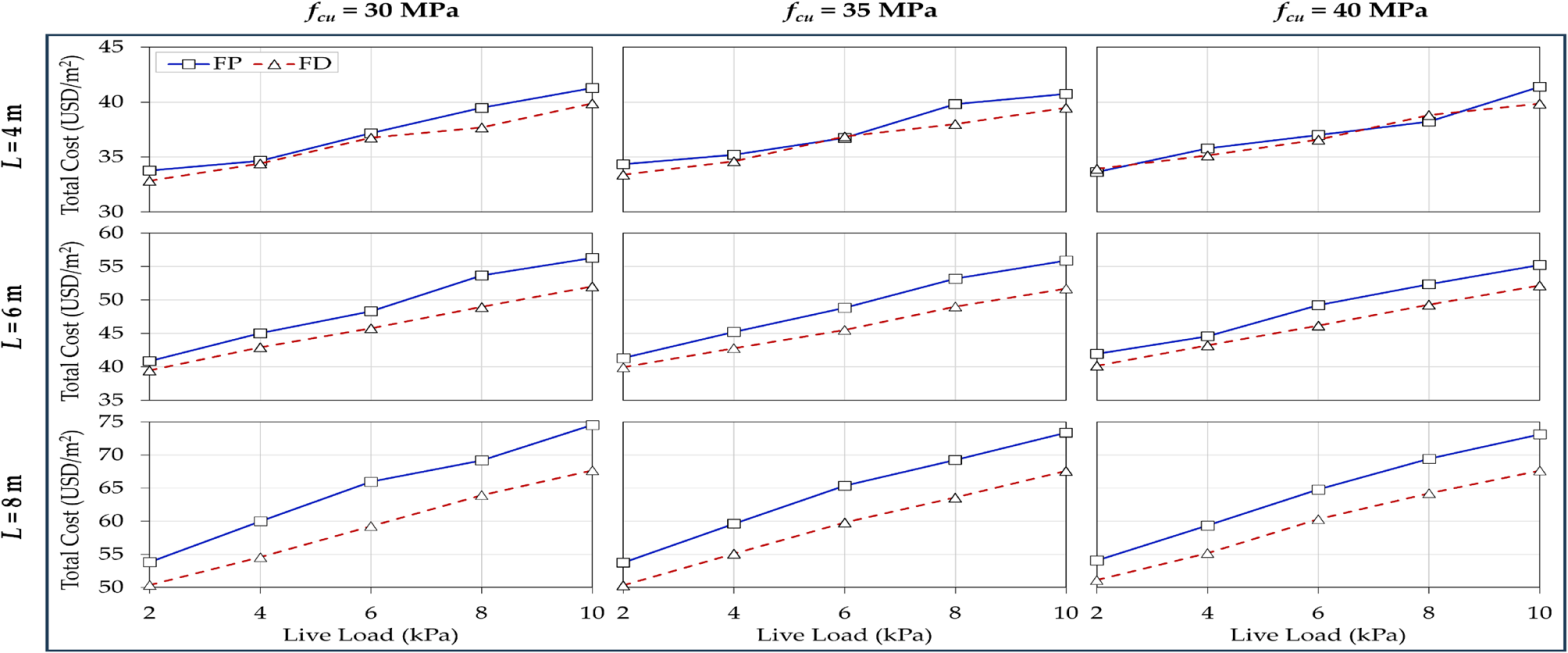
Effects of live loads on the total optimal cost for different floor systems by varying the concrete grade and spans.

Although using ${f}_{cu}$ of 25 MPa is a common practice in construction sites, it is often considered low-grade, especially for intensive usage such as office and commercial buildings. To account for potential material savings with higher concrete strength, the parametric analysis is expanded to assess the influence of the concrete grade on the optimal total cost. Figure 10 compares the impact of live loads on the total optimal cost for different floor systems, considering three higher concrete grades (${f}_{cu}$ = 30, 35, and 40 MPa).

The rows represent the span lengths, and the columns represent the concrete grades. The optimal cost ranged between 33 (50) and 75 (68) MPa for FP (FD) buildings. As expected, the total cost increased with increasing the live load for both systems. Overall, FD was cheaper than FS for different cases. For short spans (4 m), the utilization of higher concrete grades above 25 MPa resulted in substantial cost savings attributed to material reduction for both FP and FD buildings. For longer spans, higher concrete grades resulted in significant cost savings for FD buildings. The integration of drop panels and high compressive strength contributed effectively to resisting punching stresses developed from the slab-column interaction. On the other hand, insignificant changes in cost were observed with enhancing the concrete grades in the case of FP buildings. Consequently, as the span increased, the cost variation between the floor systems increased. These results tie well with the recent findings reported by Aidy et al.^3^ in the context of cost optimization of flat slab hospitals.

#### Effect of unit prices

**Figure 11:**
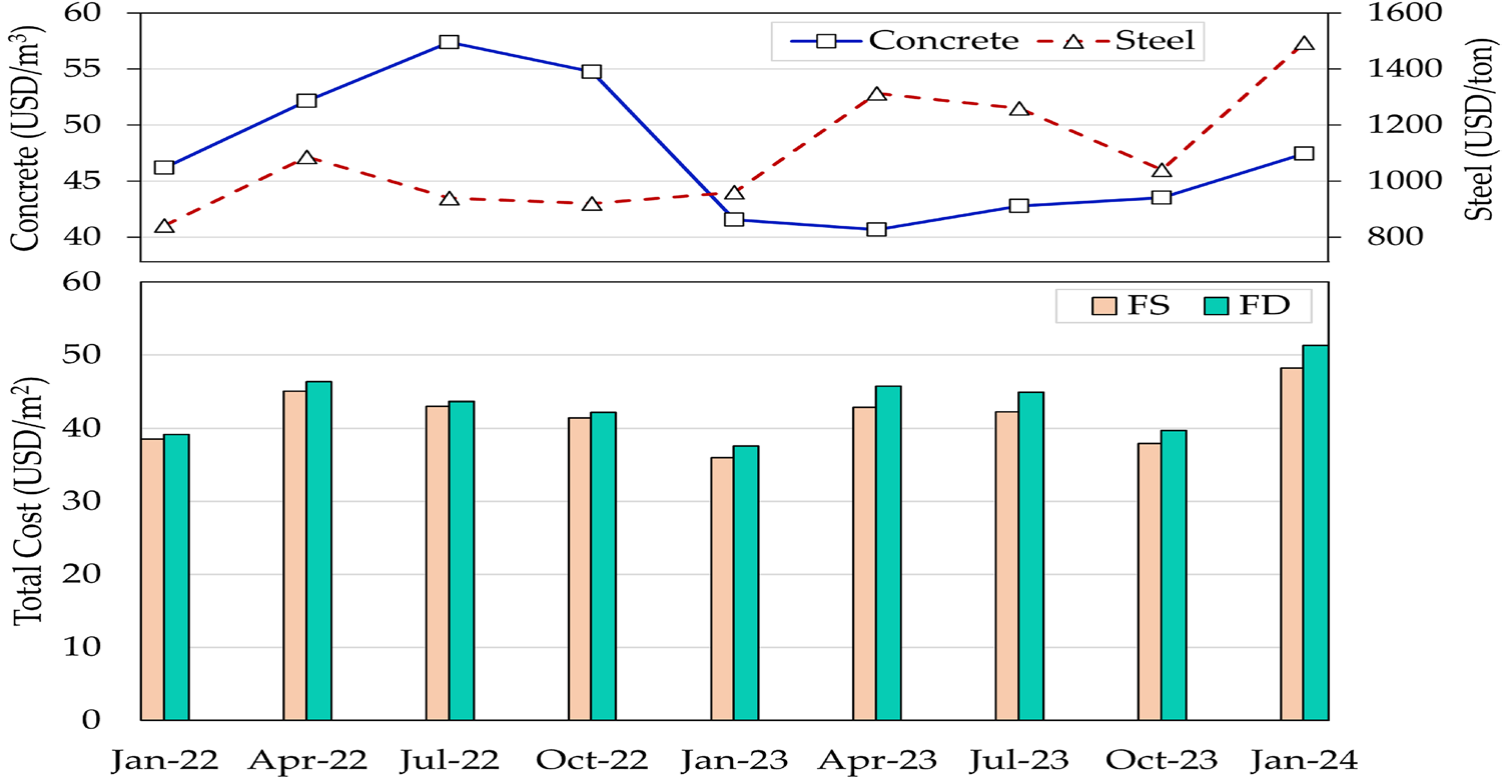
Impact of material unit prices on the optimal building cost for each floor system.

Given that the cost data was sourced for a specific period, analyzing the sensitivity of the optimized designs to price variations could provide valuable insights into the robustness and applicability of the solutions under different economic conditions. Figure 11 depicts the fluctuations in construction material pricing over the past two years in Egypt, and the impact of the unit prices on the optimal building cost for each floor system. These prices were obtained from the Egyptian monthly bulletin of material unit prices^29^ based on the local currency. The currency conversion was conducted based on the rate for each specific month. The optimal costs were calculated for flat slab buildings subjected to a live load of 4 kPa and having a span of 6 m and a compressive strength of 25 MPa. The figure reveals significant variations in material pricing. For instance, the steel unit prices almost doubled between January 2022 and January 2024. Consequently, the optimal cost for each floor system was affected by the fluctuation in material unit prices. The findings are directly in line with a recent study^14^.

#### Parameter significance

Sensitivity analysis quantifies the degree of influence that input variables exert on the desired output. One sensitivity estimation approach involves keeping a specific variable fixed while varying all other inputs^28^. Sobol’s method utilizes variance decomposition techniques to compute sensitivity indices that capture each variable's contribution^34^. Sobol’s method was employed using the R “Sensitivity” package (https://CRAN.R-project.org/package=sensitivity). The input variables considered for this analysis were the floor concrete volume, column concrete volume, floor steel weight, and column steel weight calculated for the optimal designs of different combinations of concrete grades, span lengths, and live loads for each floor system. The output parameter is the optimal cost.

**Figure 12:**
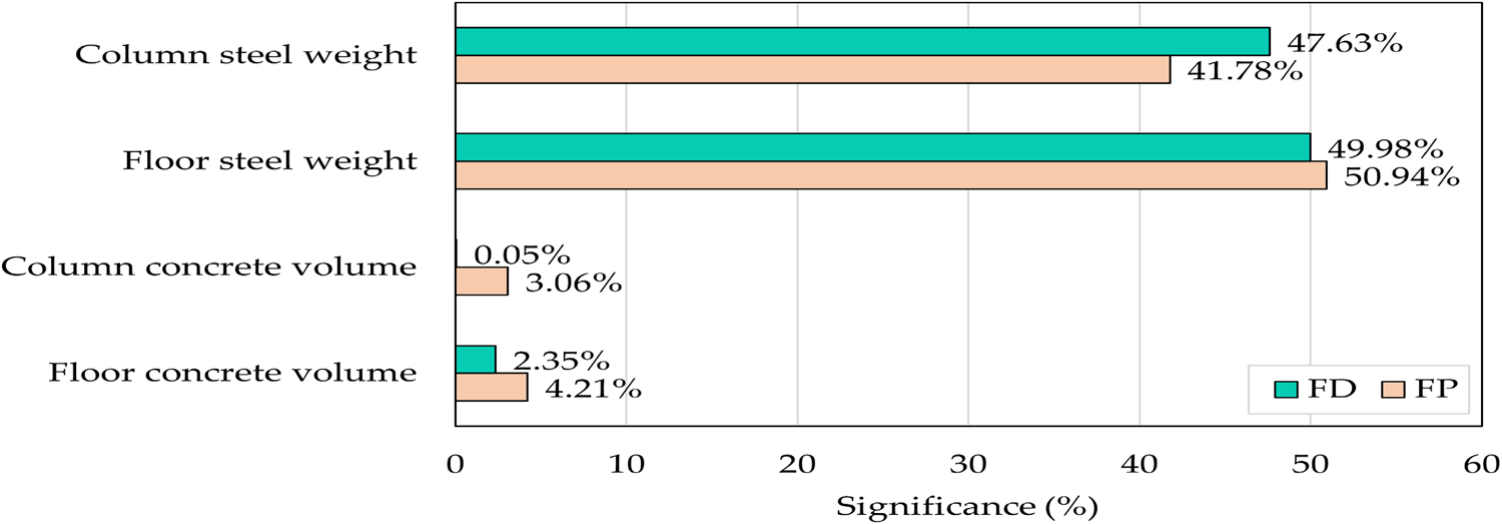
Parameter significance for each floor system.

Figure 12 illustrates the significance each input parameter on the overall cost of the building. These parameters were selected in the sensitivity analysis to capture the influence of design variables (i.e., concrete dimensions and steel reinforcement for slabs and columns) on the optimal cost of the building. The results reveal that the design variables related to steel weight contribute to over 92% of the total building cost. These results are confirmed by recent studies that attributed the reason to the significantly high steel unit prices in Egypt^3,10,14,28^.

#### Concrete volume distribution

**Figure 13:**
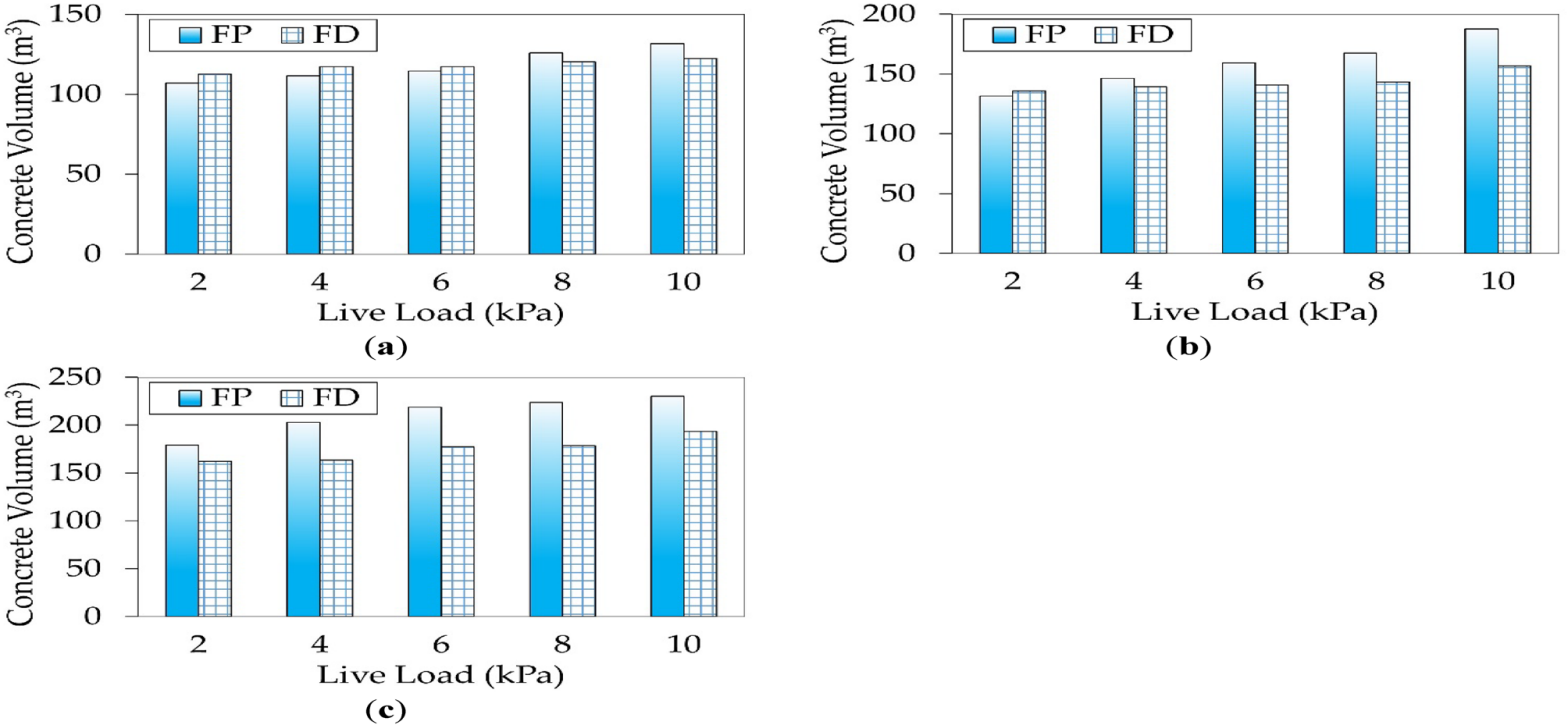
Comparison between concrete volumes for optimal designs of each floor system: (**a**) *L* = 4 m; (**b**) *L* = 6 m; (**c**) *L* = 8 m.

Figure 13 compares the concrete volumes for FP and FD buildings with ${f}_{cu}$ of 25 MPa under different live loads. For low-span buildings (4 m), FD constituted more concrete quantities up to 6 kPa because ${{t}}_{{s}}$ in both systems was 0.16 m. As the live load exceeded 6 kPa, the punching stresses of FP alternatives increased, and consequently, ${{t}}_{{s}}$ increased to 0.18 m. Accordingly, the concrete volume of FP exceeded that of FD. As the column spacing exceeded 4 m, FD efficiently resisted the punching stresses with a lower slab thickness than FP. Hence, drop panels resulted in substantial savings in the concrete volume.

#### Steel weight distribution

**Figure 14:**
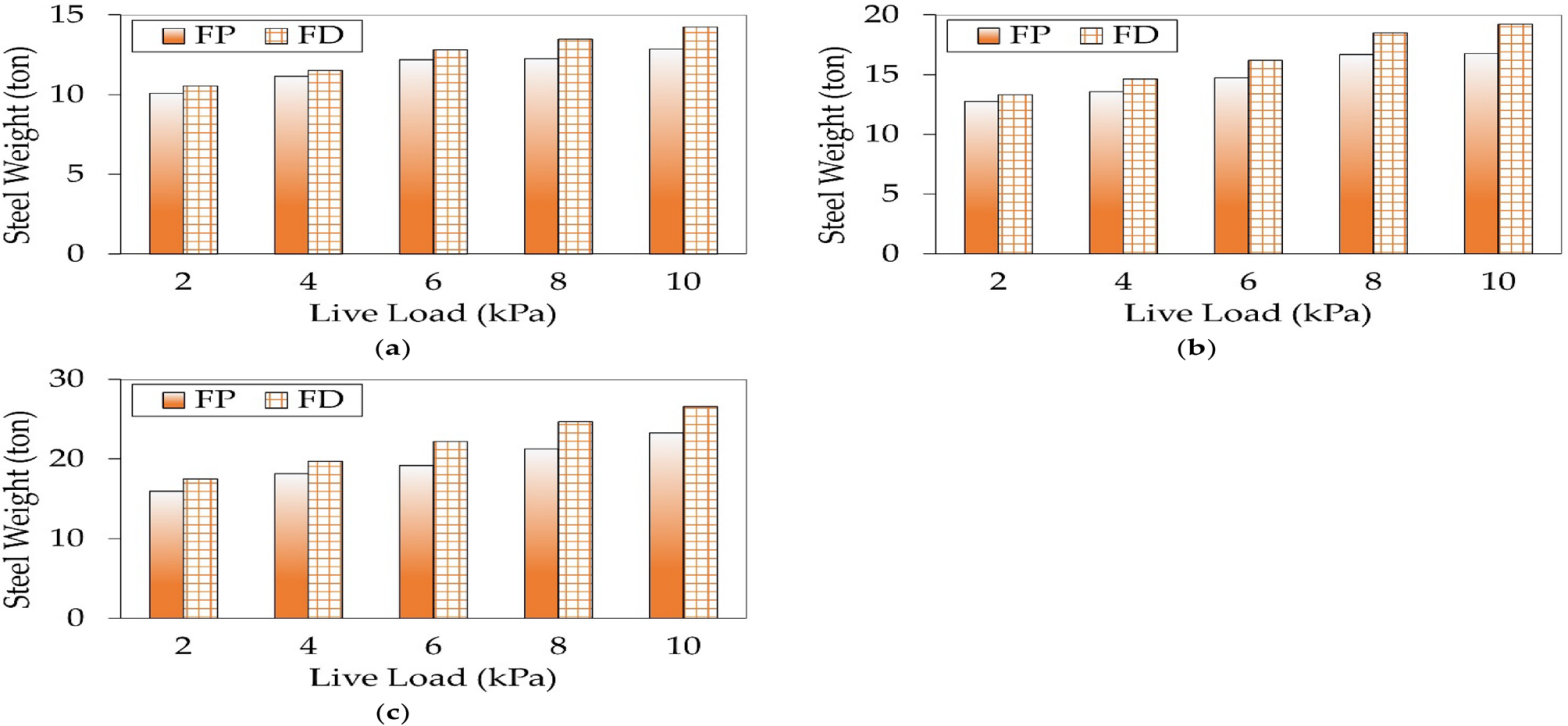
Comparison between steel weights for optimal designs of each floor system: (**a**) *L* = 4 m; (**b**) *L* = 6 m; (**c**) *L* = 8 m.

Figure 14 compares the steel reinforcement weights for FP and FD with ${f}_{cu}$ of 25 MPa buildings under different live loads. For all cases, the amount of steel in FD was higher than that of FP. Utilizing drop panels contributed to a higher flexural and punching resistance in the regions of slab-column connections. However, the steel reinforcement in FD exceeded that of FP for two reasons. First, the drop panels required a minimum amount of steel (i.e., half the reinforcement of the column strip) to fulfill the ductility requirements. Second, the effective depth decreased in the midspans, resulting in an additional reinforcement to meet the flexural capacity’s constraint.

## Conclusion

In this paper, a structural design optimization model was generated to investigate the impact of the intended function of RC flat slab buildings on their optimal cost. The model was established utilizing Microsoft Excel spreadsheets and EA provided by Solver. The author considered two floor systems (FP and FD), three column spacings (4 m, 6 m, and 8 m), and five live loads (2 kPa, 4 kPa, 6 kPa, 8 kPa, and 10 kPa). The SLS and ULS were checked for each system based on the design firm rules of ECP203-2020. The design variables were the floor dimensions and the columns’ dimensions and steel configurations. The steel bar diameters were chosen from Egyptian steel factories’ databases. The following observations were concluded:

- For spans up to 6 m, FP is economic for buildings with moderate loads, such as residential buildings, offices, and hospitals. However, FD is cheaper than FP for structures with high column spacings (8 m), regardless of the building’s function.
- As the live load increases, the cost difference between the floor systems decreases.
- Enhancing the concrete compressive strength resulted in reasonable cost savings attributed to material reduction only in the case of FD buildings.
- For buildings with low live loads (i.e., residential buildings) and column spacings (4 m), FD requires a higher concrete volume than FP due to the undue utilization of drop panels. As the live load and column spacings increase, a significant reduction in concrete volume is observed for FD.
- The total steel reinforcement weight of FD buildings is higher than that of FP, regardless of the building’s function and column spacings.
- For all optimal designs, the optimizer tends to utilize a low reinforcement ratio for columns to reduce the overall cost of the building.

The findings from this optimization study have significant implications for the broader field of structural engineering. The present study provides practical guidelines for the designers to choose the adequate floor system based on the intended function and range of spans for flat slab buildings. While this study focused primarily on cost optimization for flat slab buildings based on expenses of labor and materials, future work should explore multi-objective optimization formulations that simultaneously minimize both financial costs and embodied carbon emissions from material production, enabling more holistic sustainability assessments. Furthermore, the influence of energy consumption on the total cost was not assessed in the present scope. Thus, building performance simulation could be employed using weather meteorological parameters and building envelope. The author assumed that the unit prices of labor and formwork are the same for both floor systems. However, accurate unit prices of labor and formwork could be evaluated to account for the complex installment of drop panels on the construction site. Since ambiguities and uncertainties exist in cost data or assumptions, employing neutrosophic statistics may help address uncertainty and improve optimization outcomes. Extending the current optimization approach using neutrosophic statistical techniques represents a promising direction for future research to further enhance cost minimization for RC flat slab structural design. Because the structural design was based on the guidelines of the Egyptian code of practice, it is crucial for engineers to carefully analyze and account for the key distinctions between relevant design codes to deliver adequate structural designs. It is important to note that the current study did not consider seismic loading in the optimization model. Future research should extend the proposed approach to incorporate seismic design considerations, which are crucial in regions prone to seismic activity.

## Data Availability

The data that support the findings of this study are available on request from the corresponding author.
